# Types of Utilization and Types of Treatment Response in a Collaborative Care Approach for Depressive Disorders in Old Age in Primary Care

**DOI:** 10.3389/fpsyt.2020.565929

**Published:** 2020-10-23

**Authors:** Thomas Kloppe, Nadine Janis Pohontsch, Martin Scherer

**Affiliations:** Department of General Practice and Primary Care, University Medical Center Hamburg-Eppendorf, Hamburg, Germany

**Keywords:** depression, collaborative care, qualitative study, mixed methods analysis, primary care, typology, health care research, Germany

## Abstract

**Background:** Treatment of depressive disorders in old age is hindered by several barriers. Most common are time pressure in primary care and latency for specialized therapeutic care. To improve treatment, the collaborative care approach GermanIMPACT was evaluated in a cluster-randomized controlled trial. Care managers offered a complex stepped-care intervention of monitoring, psychoeducation, and behavioral activation techniques. Twenty-six percent of the intervention group responds with a remission of depressive symptoms compared with 11% who received treatment as usual (TAU). The low-threshold intervention was more successful than TAU. Nevertheless, three-quarters did not respond with a remission. The aim of this study is to identify and describe the different types of utilization and of treatment response to understand what constitutes an effective intervention.

**Methods:** Of 64 patients from the intervention group, we carried out problem-centered interviews with 26 patients from the intervention group. We analyzed the interviews using a qualitative type-building content analysis. For type construction, we performed a contrasting case comparison, regarding inductive and deductive categories of the intervention utilization and the symptom development.

**Results:** The 26 participants' ages ranged from 62 to 87 years (mean = 72 years). Three participants were male. We identified five types of utilization, which differ primarily in the realization of pleasant activations, depending on own activity at the beginning and during the therapy: “activatable relief seekers,” “active relief seekers,” “active relaxation seekers,” “passive problem-solving seekers,” and “passive relief seekers.” In the second typology, we analyzed four deductively determined types of treatment response *responders, slight improvers, constant moderates*, and *non-responders*. Patient-specific characteristics are a recent history of depression, an affinity for activities, supportive contacts, and limited comorbidity. In contrast, non-responders report contrary characteristics.

**Conclusion:** Our two typologies emphasize that an effective intervention requires a match between intervention components and patient characteristics. We saw no intersections between utilization and treatment response. GermanIMPACT is an effective low-threshold intervention for moderately burdened patients, who are still capable of self-activation. An expansion of the intervention, especially for depression with a long history and comorbidities impairing mobility, could increase the effectiveness and improve the care situation of older people suffering from depression.

## Introduction

Depression is the largest single cause of disability worldwide (1) that affects one in five people at least once in their lives (2). In Germany, one in seven individuals is affected by depression within 1 year (3). Depression impairs the quality of life and increases the risk of diabetes mellitus type 2, coronary heart disease, chronic pain, and other geriatric syndromes (4). German health care costs caused by persons with depression are almost twice as high as those of non-depressive patients (5). At the same time, depressive disorders are associated with high social costs. Krauth et al. (6) estimate the annual costs per patient with depression at 3.813 euros, which represents a total cost of 15.6 billion euros in Germany.

In German primary care, depression is the most common mental disease (7). The treatment of depressive disorders in old age is effective (8) but hindered by several barriers. Older patients avoid specialized care because of the risk of self-stigmatization (9, 10), as well as their own negative age-related attitudes (11). At least 50% of affected patients remain exclusively in primary care because they prefer it this way (12, 13). Resources are very limited; the greatest challenge is time pressure (14).

In order to alleviate the burden of general practitioners (GPs), the US-American collaborative care approach IMPACT was adapted to the German health care system, and “GermanIMPACT” was evaluated in a cluster-randomized controlled study in Hamburg and Freiburg (15). In this nurse-led intervention, care managers offered patients a low-threshold telephone intervention with a stepped care approach. The control group received treatment as usual (TAU) by GPs. This complex intervention, which was carried out over 1 year, was superior to TAU; 25.6% of the intervention group showed a remission of depressive symptoms compared with 10.9% of TAU (16). This result is comparable with the original US study (17), as well as with other international adaptations (18). Nevertheless, three-quarters of the patients in the intervention group did not respond to the therapy support and did not achieve a satisfactory reduction in depression.

We saw that self-reported engagement in activities and depression outcomes are linked (19), and we assume that the further elements of the stepped-care approach worked in various ways, depending on the type of individual utilization (20). However, neither the previous US study nor other studies have been able to explain how the individual utilization should be valued and why some patients respond to treatment and many patients not (21).

To increase the effectiveness of the IMPACT intervention, it is important that we understand which intervention components are needed for which patient type. The aim of this article is the identification of patient types to evaluate factors that facilitate or hinder the ability to respond to the GermanIMPACT treatment.

The research questions are as follows:

1. Which types of utilization could be identified?
2. Which types of treatment response to the intervention can be identified and what are their specific characteristics?
3. Which patient-specific characteristics of the types of treatment response could be identified in types of utilization?

## Methods

To evaluate the cluster-randomized controlled trial GermanIMPACT (German Clinical Trials Register identifier: DRKS00003589, funded by the Federal Ministry of Research and Education) and to investigate the subjective perspective of patients with late-life depression, we performed an “explanatory sequential design” (22) with a qualitative comparison of cases in a nested qualitative interview study following the controlled intervention trial. The faculty of the University Medical Center Hamburg-Eppendorf pays the open-access fee for the journals in the *Frontiers* series.

### Ethics Statement

This study received ethical approval from the Ethics Committee of the Medical Association of Hamburg on 10 June 2013, reference number PV4480.

### Study Setting—The GermanIMPACT Cluster-Randomized Controlled Trial

In 71 general practices in Hamburg and Freiburg, during their consultation hours, the GPs asked all men and women 60 years or older who had moderate depressive symptoms to be screened for depression with the Patient Health Questionnaire-9 (PHQ-9). Patients with a PHQ-9 score between 10 and 14 points and without substance dependence disorder or pronounced cognitive impairment (e.g., dementia) or ongoing psychotherapy were asked for a written consent to participate in the study. Two hundred and forty-eight patients have been included; 139 patients were randomized to the intervention group, and 109 patients to the control group. In the intervention group, care managers offered a complex stepped-care telephone intervention.

Main components of this intervention were monitoring, psychoeducation, and behavioral activation. At first, patients should learn what constitutes depression, what symptoms can occur, and what they can do against them. Primarily, they should learn to monitor their symptoms by using PHQ-9 to get a feeling for their mood changes, and most importantly, they should be encouraged to enjoy pleasant activities to feel better. In further steps of the stepped-care approach, it was possible to get advice on medication and six special lessons to learn problem-solving techniques. The extended collaborative care team consists of GP and a supervising psychiatrist or psychotherapist.

The control group received TAU. The primary outcome was the change of the PHQ-9 score according to the close US-American IMPACT study (17) and a comparable German trial (23). Following Kroenke et al. (24), response to treatment was defined as a symptom reduction of 50% or more, and remission was defined as a PHQ-9 level below 5. After 12 months, the adjusted and estimated remission rate in the intervention group was 25.6% (95% confidence interval, 18.3–32.8), correspondence, 10.9% (5.4–16.5) in the control group (*p* = 0.004). The detailed study design and the main outcomes are described elsewhere (15, 16). The rate of response to treatment was even higher in the intervention group, 22.5% (14.6–30.5) compared with 10.5% (4.1–16.9) in the control group.

### Data Collection

#### Participant Selection and Recruitment

**Figure 1:**
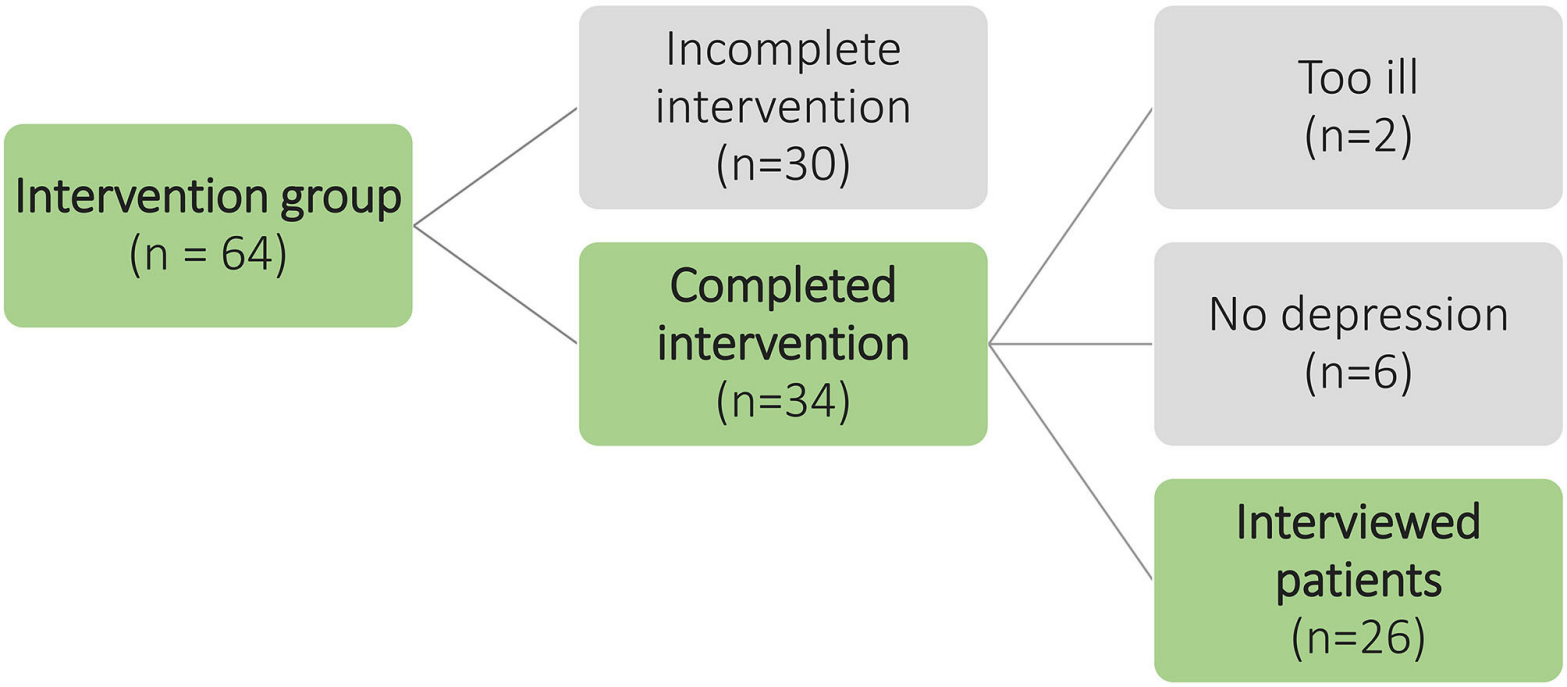
Flowchart participant selection and recruitment.

In Hamburg, 64 patients received the intervention. Thirty-four patients completed in the regular period of 12 months. Thirty patients terminated the intervention prematurely, because they supposed that they were not requiring depression treatment, their health condition improved, their personal effort seemed too high, they were more concerned about other physical diseases, or they thought the therapy support is not helpful. We invited 34 potential interviewees to an interview. Two patients declined to participate because their state of health had deteriorated considerably. During the preliminary interview, we discovered that six patients had been incorrectly enrolled in the study because of a false-positive PHQ-9. They did not suffer from depression, but they appreciated the conversations with the therapy support and completed the intervention. Finally, 26 patients were recruited who completed the intervention and suffered from depression. For an overview of the selection and recruitment of participants, see Figure 1.

#### Allocation to the Types of Treatment Response

The depressive symptoms, measured with PHQ-9 (25) after finishing the GermanIMPACT intervention, ranging from a score of 1 to 25 out of 27. Following the primary outcomes of GermanIMPACT, nine of 26 patients were categorized as *responders* because their depressive symptoms, measured with PHQ-9, halved (response to treatment) or their score felt below 5 (remission). Ten participants constituted the type of *non-responders* because their PHQ-9 score was constantly above 10. The differentiation into responders and non-responders proved to be ineffective in the qualitative content analysis. Only one point separated patients 10, 12, 16, and 17 from responders. The discrimination power of the quantitative study could not be confirmed empirically in the present qualitative study. Therefore, we formed two new groups to represent the transition area between responders and non-responders and to allow a more differentiated typology of symptom development. Four patients constitute the type *constant moderates*. Those patients started and ended the trial with a low PHQ-9 score of 5 or 6 and did not change throughout the 12-month observation period. Further three patients were assigned to the second new group: *slight improvers*. They were able to reduce their score by three points after 1 year, achieved a score of 10 or less, and judged the therapy support to be effective. For an overview of the allocation, see Table 1.

**Table 1:** Age, gender, and classification of responders.

**Patient characteristics**	**Female**	**Male**	**Age ≤ 75 years**	**Age ≥ 75 years**	**Total**
Female	23	—	15	8	23
Male	—	3	1	2	3
Age <75 years	15	1	16	—	16
Age >75 years	8	2	—	10	10
Responders	8	1	7	2	9
Non-responders	9	1	5	5	17
Constant moderates	4	0	2	2	4
Slight improvers	2	1	2	1	3
Total	23	3	16	10	26

***Responders**: Patients in remission [score <5 on the Patient Health Questionnaire (PHQ-9)] or patients with a reduction of symptoms by at least 50% (response to treatment) at the end of intervention, 12 months after baseline*.

***Constant moderates**: Patients with a PHQ-9 score of 5 or 6 at the beginning and after completing the intervention*.

***Slight improvers**: Patients who reduce their symptom score by three points after 1 year and who achieved a score of 10 or less*.

***Non-responders**: Patients without a reduction of symptoms and without a score <10*.

#### Problem-Centered Interview

To explore people's subjective experiences, we carried out semistructured, problem-centered interviews. This method of interviewing developed by Witzel and Reiter (26) and Witzel (27) attempts to combine theory guidance and openness by enabling the subjects to present their views and their perspective on the disease in a comprehensive and interactive manner. The interviewees are supposed to use their specific knowledge and ask questions that are concretely focused on the problem. The interview situation in the problem-centered interview is a hermeneutical process. Interviewers must bring the subjective aspects and theories of the interviewees in line with their own patterns (deduction), and they must be able to abstract new patterns inductively from the individual phenomena. In particular, the interview participants should experience a real conversation situation through “requests for narration,” “general and specific soundings,” “factual questions,” “reflections,” “questions of understanding,” and “confrontations,” so that a “discursive generation of understanding and material” is possible.

The interview guide was based on two narrative-generating stimuli:

1. What do you think, why did the doctor recommend the study to you?
2. Please describe to me how the therapy support program worked for you. I would like to imagine how it worked out for you. Tell me about it from beginning to end.

The interviewer was challenged to intervene, ask questions, reflect, refocus, or ask for interpretations. To shape deeper inquiries (28) and to ensure a comparable level of saturation, we used a checklist of topics and several pre-formulated follow-up questions.

#### Setting

A total of 26 interviews were conducted between January 2015 and June 2016. To achieve a maximum of openness, the interviewer was blinded to the patients type of treatment response.

All interviews were conducted and audio-recorded by the first author. Ahead of interview, a detailed study information was handed out to each participant. Full informed written consent was obtained by the interviewer before starting the interviews. The length of the interviews varied between 28 min and 1 h 42 min; the average duration was 56 min. Twenty-one interviews were conducted with the interviewees at their home, two at the interviewer's office and three by telephone.

### Analysis

All interviews were transcribed verbatim with a slight smoothing (29) using the software f4 (30), and we performed all analyses by using the software MAXQDA 12.1 (31).

To reorganize the data, we used the “multistage model of empirically grounded type construction” (32). This approach attempts to construct “real types” based on three steps.

1. In a qualitative content analysis (33), we concentrated the huge amount of data. We derived the main categories deductively from the two impulse questions of the interview guide. Subcategories were built inductive. Additionally to the transcript, we used field notes after the interviews, notes during the analysis, oral peer debriefings, and discussions about the coding framework in an interdisciplinary working group (33). In addition, we created structured case vignettes for all interviews, which helped to identify and organize systematically the relationships between categories, codes, and memos (26).
2. We carried out a contrasting case comparison across all developed categories. These comparison of all patients showed three categories, which allowed a systematic differentiation between the patients (32).
3. Finally, we performed a so-called scaling and evaluating content analysis (34). In this step, we condensed and scaled similar subcategories using a clear dimensionalization. As shown in Table 2, we assigned all specific codes to a few manifestations and arranged them hierarchical. To identify different typologies, we interpreted and scaled inconsistent patient statements against the entire interview, including the developed case vignettes.

**Table 2:** Framework of the scaling, evaluating content analysis.

	**Categories**	**Dimensionalization**	**Manifestation**	
	Pleasant activations	How does the patient evaluate the core element of the therapy support and especially its implementation?	++ + – −−	Successfully implemented Already active in the beginning, relaxation techniques have been implemented Rejected, patient was already active Realization did not work
Types of utilization	Emotional relief	How important this aspect was for the patients?	++	High important value
	Counseling on problems	How does the patient evaluate the support provided by counseling in case of problems?	+ – −−	Has worked Has not taken place Has not worked
	History of depression	How does the patient explain the onset of depression?	+ – −−	Time-limited recent depression trigger Distinct depression trigger more than 10 years ago Depressive symptoms already exist for a longer time, the trigger cannot be defined
	Burden of symptoms	How does the patient describe his/her burden of symptoms?	+ –	Limited disease burden High disease burden
Types of treatment response	Activity prior to intervention	How does the patient describe his/her own activity before the intervention?	++ + –	Strong Moderately Quite low
	Comorbidity-related mobility	How does the patient describe his/her mobility in the face of comorbidities?	++ + –	High mobility Possible with restrictions Immobile
	Self-confidence	How does the patient describe his/her self-esteem and self-efficacy?	++ + –	Strong self-esteem Ambivalent statements on self-esteem Very insecure until bad opinion of oneself
	Social inclusion	How does the patient evaluate his/her social inclusion?	++ + –	Trustworthy supporting contacts Few contacts/support Sad about the lack of contacts

## Results

A total of 26 interviews were conducted between January 2015 and June 2016. The age of the participants ranged from 62 to 87 years, with a mean age of 72 years. Three participants were male. We developed (1) five types of utilization and (2) four types of treatment response. In a third step (3), we examined both typologies together.

### Types of Utilization

The five identified types of utilization are as follows: *activatable relief seekers, active relief seekers, active relaxation seekers, passive problem-solving seekers*, and *passive relief seekers*. They were based on various manifestations in three constitutive categories: *pleasant activations, counseling on problems*, and the type-spanning category *emotional relief*. These manifestations are expressed in a certain degree of activity. That is why we developed five type labels with the proactive term “seeker.” It emphasizes the self-active, striving, and demanding character of patients who belongs to the types of utilization. As shown in Table 3, this type benefited in different degrees from the constitutive elements of the intervention, depending on their own activity.

**Table 3:** Types of utilization based on three constitutive categories.

**Type of utilization**	**Pleasant activations**	**Emotional relief**	**Counseling on problems**
Activatable relief seekers Patient 2, 5, 6, 11, 13, 18, 20, 22, 26	Were able to plan and implement activities	Were able to benefit from the emotional relief	Were able to benefit from Counseling on Problems
Active relief seekers Patient 8, 9, 12, 17, 19, 21	Have already implemented all the activities they considered important		
Active relaxation seekers Patient 1, 3, 10, 14, 15	Sought and planned conscious pauses for relaxation		Could be activated with problem solving strategies
Passive problem-solving seekers Patient 7, 23, 25	Could not identify and implement any Pleasant activities		
Passive relief seekers Patient 4, 16, 24			Could not benefit from the Counseling on Problems

Each patient could be assigned to one of the five types of utilization. First, the patients differed primarily in the way of using the element pleasant activations. In the analysis of the interviews, we saw that patients could only use the intervention components for themselves if they were able to show a little self-initiative.

### Activatable Relief Seekers

The *activatable relief seekers* described a high level of utilization and the highest benefits in the seeking and implementation of pleasant activities. “Activatable” refers to the capability of benefiting from the element of activation and the common planning of pleasant activities. Starting from a low level of activity, patients of this type were able to integrate pleasant activities into their everyday lives and apply them constantly.

“And she also suggested that I should do this and that, and flowers, and plants and so on. And yet I thought, that was good. So, what Mrs. ^***^ had said and asked and suggested, I thought, was very good.”

(Patient 2, >75 years, non-respondent, par. 80)

Even the correspondence aspect of control was described positively. By discussing the planned activities, the participants felt forced to implement these activities and to increase their own activity:

“Yes, for me it was OK, good. I always wanted to keep my promise, I say, Mrs. ^***^, I did not succeed. I didn't do it, but I did, yes.”

(Patient 22, <75 years, slight improvers, par. 122)

In most cases, even the element of *Counseling on Problems* was described in a positive way:

“For example, when I have something to cope with, right? Then I always thought, oh God, you'll never manage that in your life again, right? How do you want to do all that? Then I heard from Mrs. ^***^, I have stored everything correctly in my head, ‘Why don't you take half an hour first and then leave it all there again and then start it again.' It still doesn't work always, but I know I can fall back on it and put it into practice.”

(Patient 20, <75 years, non-respondent, par. 76)

Even more than Counseling on Problems, the *activatable relief seekers* reported about the emotional relief, they experienced in conversations with the care managers. The patients reported different beneficial effects of mirroring and the reinterpretation of daily situations that enabled them to break through cycles of negative thoughts:

“If my daughter doesn't call for a long time, long, that's two or three days maybe, then I already think, ‘What have I done wrong? Why she doesn't care?' That she has no time, that comes only in the second train of thought.”

(Patient 6, >75 years, non-respondent, par. 250)

The emotional relief includes everyday concerns that determined the thoughts of the patients. They were not able to have this kind of conversation with their relatives or friends. That is why the therapy support was a great enrichment from their point of view:

“It doesn't have to be so productive every time, so it can just be that you can cry on someone's shoulder. And what is also important, that this person has nothing to do with the whole situation.”

(Patient 13, <75 years, respondent, par. 62)

### Active Relief Seekers

The second type *active relief seekers* used the component of emotional relief as well.

“Well, I had confidence in her, and we talked on the phone quite often, about everything and I must say, it's always been good to get a lot off my chest.”

(Patient 19, <75 years, respondent, par. 136)

Other components of the GermanIMPACT intervention were barely used by this type of patients. But the primary difference between this type and the *activatable relief seekers* was the handling of *pleasant activations*. Patients of this type described themselves as active enough and denied extra planning of pleasant activities.

“I mean, she has some suggestions, that was really good, but as I said, I think, I should not tie any more to my leg, so that's what I'm busy with. When I have more time or I don't know, maybe in winter or, I don't know.”

(Patient 21, <75 years, non-respondent, par. 104)

### Active Relaxation Seekers

The third type, the *active relaxation seekers* showed a need for conscious breaks. These patients planned well-defined relaxation phases to break up the structure that pressured and overtaxed them. One recently retired patient got depressive symptoms, because of her claim to structure and fill up every day completely, as she did it in her working life. She described the name-giving elements of the type active relaxation seekers in a visual way:

“[.] I didn't put myself under so much pressure anymore, that I have to be there every hour now, you know? It can also be a little more relaxed (2), that is also quite pleasant.”

(Patient 15, <75 years, respondent, par. 67)

Patients of this type used almost every GermanIMPACT intervention component except pleasant activations.

### Passive Relief Seekers

In contrast to the previous types, the *passive relief seekers* were not able to choose, plan, and perform pleasant activities during therapy. The patients said that their time was over, that they felt physically too limited, or that they simply could not motivate themselves.

“She's always tried to make it, she always tried: Mrs. ^***^ what are you having fun with? But I must realize, I must be willing to do that. And then I say ‘yes' in that moment and then I think, ‘Nooo. That's it.”

(Patient 7, <75 years, Slight Improver, par. 138)

The GermanIMPACT intervention overwhelmed them, and the low level of activity in this type partly resulted in an even stronger desire for emotional relief:

“Yeah, that was nice, wasn't it? That was (2) As I said, I've become so shy of people, so at the moment I can't be with neighbors either, I can't, uh (2), I don't know, somehow, I've become strange. I don't know.”

(Patient 4, >75 years, non-respondent, par. 177)

### Passive Problem-Solving Seekers

Contrary to the extremely low utilization by the *passive relief seekers*, the *passive problem-solving seekers* had a medium intensity of utilization. The term “problem solving” refers to an increased self-activity regarding the handling of problems. Patients of this type could be activated with mediated problem-solving strategies. Nevertheless, these problem-solving activities cannot be regarded as pleasant activities, but they have a similar mood-enhancing effect for the patients:

“Cleaning up papers, I have everything in files, not even neatly [.] and then in the next room everything lays on the floor. I described it all to Mrs. ^***^, who wasn't here. Then I said, ‘That has to go away.' Then she said, ‘When you make 10, you look at 10 sheets.' I said, ‘no, if I do something, I do it all.' Then she said, ‘That won't work. Do 10, look at 10 and then do 20.' I did it. It's all gone.”

(Patient 25, >75 years, non-respondent, par. 188)

### Types of Treatment Response

The categorization of the types of treatment response is deducted from the change in depressive symptoms (PHQ-9) as a response to the GermanIMPACT intervention: *responders, non-responders, constant moderates*, and *slight improvers*. In the following, we describe the six inductively developed categories and how they differ within the types of response.

### History of Depression

A common characteristic of the *Responders* and the *slight improvers* is the determinable trigger of depression in recent history. The patients described recent conditions of overstrain and helplessness, for instance, related to the loss of a family member. They report long periods of informal caregiving, own diseases, broken marriages, and their retirement as depression triggering factors.

In contrast, patients of the types *non-responders* and *constant moderates* reported less recent but similar triggers 10 to 20 years ago: the death of a spouse, prolonged chronic pain, or the loss of their job. Partly they report various (inpatient) therapy experiences and partly a general “predisposition” for depression:

“Well, I guess, started in my childhood. But I was always able to cover it up because it was so early in my life. […] nobody should notice.”

(Patient 5, >75 years, non-respondent, par.75)

### Perceived Burden of Symptoms

All patients reported similar symptoms of depression: feeling down, social withdrawal, hypersensitivity, changes in behavior, brooding cycles, insomnia, and anxiety. But in contrast to the *non-responders, responders, slight improvers*, and *constant moderates* assessed their perceived impairment as not that high and manageable:

“I always have two choices, either, ‘I'm sorry, I'm sad, I'm a mess,' or ‘I'll make the best of it.”'

(Patient 9, >75 years, non-respondent, par. 58)

*Non-responders* mostly suffered under a high burden of symptoms, which already existed over a long period of time. Even if they had an idea how they might feel better, they still described a strong apathy that impaired them:

“I don't have that stamina. I miss it.”

(Patient 7, <75 years, slight improver, par. 188)

### Comorbidity-Related Mobility

Even symptoms of mental comorbidities such as anxiety and agoraphobia were described as a depression trigger.

“Sometimes I was afraid to go out alone. [.] That something happens to me on the way and there is nobody to help me. That was the main problem.”

(Patient 16, <75 years, slight improver, par. 20)

All interviewees were burdened with somatic comorbidities, such as diabetes mellitus type 2, high blood pressure, bronchial asthma, arthrosis, rheumatism, condition after stroke, and condition after breast cancer, as well as tonsil cancer and condition after operated spinal canal stenosis.

Compared to *non-responders, responders* largely felt that they could cope their comorbidities and that they were “well adjusted” medically. They usually described good mobility, which enabled them to do activities in the city, do sports, and to travel. *Non-responders* described severe somatic complaints, which did not allow them a way out of their passivity.

### Activity Prior Intervention

Most *responders* already described themselves as active at the beginning of the intervention and formulated no need for activation. They visited sport courses, went jogging or walking, went on holiday, went to concerts or theater, they read books, met friends, and much more. In most cases, the *responders, constant moderates*, and *slight improvers* understood activity as a good coping strategy against depressive symptoms.

“Yes, I do sports. [.] So I move a lot. And that's good for me. Fresh air, I need a lot of fresh air.”

(Patient 14, <75 years, respondent, par. 56)

### Social Inclusion

Mostly the level of activity and the integration into social contexts were interdependent and influenced their symptoms. *Responders* mainly reported about a solid social network with communicative exchanges and support. *Non-responders* were not in familiar bonds, and even if they tried to make personal contacts, they often failed to overcome their loneliness. Nevertheless, nearly all patients thought that they had to cope independently with symptoms of depression like the lack of interest. They did not experience the relationship with relatives as a shelter for intimate conversations, rather as a shelter in which the relationship should not be disturbed.

### Self-Esteem

*Responders* described their employment biographies predominantly as positive and meaningful, giving chance to build up a generous amount of self-esteem. *Responders'* statements indicate a rather stable, resilient personality. In contrast, *non-responders* described many moments of uncertainty in their lives, in their everyday activities, in the use of help in general, as well as in therapy support in particular:

“[.] that was such a big help this year, that really was a big help. […]. First (2), the doctor, he put me there, I don't take such a step alone.”

(Patient 20, <75 years, non-respondent, par. 268)

In summary, the four types of treatment response are characterized by six constitutive categories. Their specific configuration is shown in Table 4. Responders have a more recent and determinable history of depression, a personal confidence in dealing with existing depressive symptoms, a bigger activity, a lower burden of comorbidity, a higher mobility, a higher self-esteem, and a higher social integration as *non-responders*.

**Table 4:** Schematic illustration of the types of treatment response.

**Type of treatment response**	**Responders**	**Slight improvers**	**Constant moderates**	**Non-responders**
History of depression	Determinable trigger in the recent past		Recurrent relapses, over 10 years	
Perceived burden of symptoms	Accepted and changeable	Low, but difficult to change		High, unchangeable
Comorbidity-related mobility	Good mobility despite comorbidity			Severe constraints
Activity prior intervention	Hobbies, holidays, responsibilities		Little	Inactive
Social inclusion	Solid social network			Loneliness/burdeningrelationships
Self-esteem	High		Low	

### Utilization Types and Types of Treatment Response

**Figure 2:**
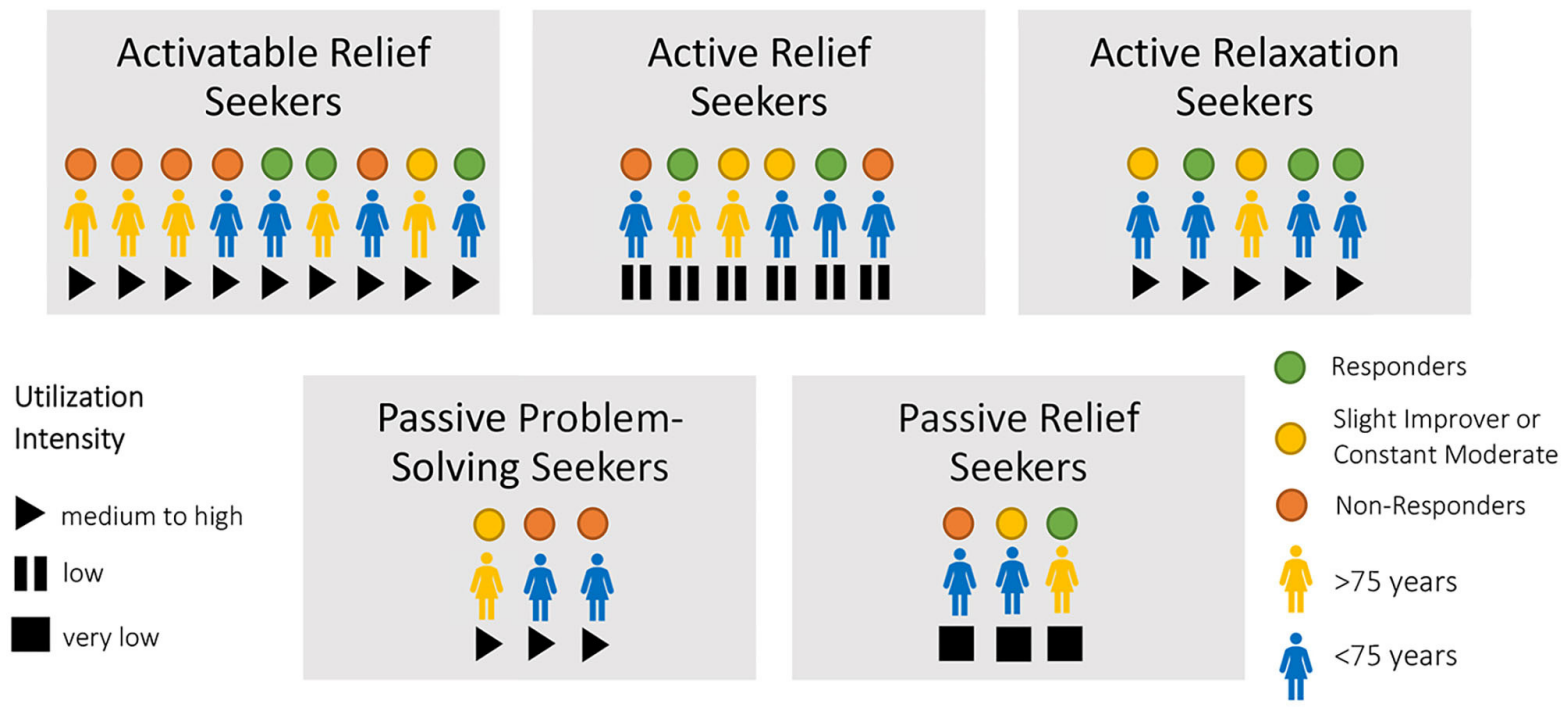
Illustration of the utilization types regarding utilization intensity and effectiveness categorization.

Patient-specific characteristics of the types of treatment response could not be identified in types of utilization. The type of utilization does not seem to determine an effective intervention measured with PHQ-9. Figure 2 shows the patients within the types of utilization regarding their intensity of utilization and regarding their type of treatment response into *responders, slight improvers, constant moderates*, and *non-responders*.

All types of treatment response are distributed over all types of utilization. This variation shows that the patients who benefited most from the central component pleasant activations—and who also reported high intensity of utilization in general—rarely became *responders* to the study.

## Discussion

In this qualitative comparison of cases, we identified patient types, and we uncovered and evaluated patient-specific characteristics that facilitate and hinder the ability to benefit from the intervention in GermanIMPACT.

1. The distinguishing characteristics in the types of utilization are the ability to use the three intervention elements: pleasant activations, counseling on problems, and emotional relief.
2. The types of treatment response (categorized with PHQ-9 measurement) depends primarily on the presence of a determinable depression trigger in the recent past, of a moderate burden of symptoms, and of the presence of a good mobility, a limited comorbidity, a general interest in activities, a solid social network, and a good description of their self-esteem.
3. We found no patient-specific characteristics of the types of treatment response within the types of utilization. Only patient-specific characteristics described within the types of treatment response seem to constitute the effectiveness of the GermanIMPACT intervention. The different types of utilization do not suggest that they have an impact on the effectiveness of the intervention.

### Types of Utilization

There are parallels to the types of utilization in this study described by Bennet et al. (35). In their trial of “Pro-Active Care for Chronic Depression” they identified two types of utilization: the “engaged patients” and the “reluctant patients.” The first type was motivated to change their situation like the *activatable relief seekers, active relief seekers, active relaxation seekers*, and *passive problem-solving seekers*. In contrast, the “reluctant patients” showed a similar behavior as the *passive relief seekers*. Because of their fatalistic perspective, it was impossible to motivate this type for methods such as pleasant activations or other techniques of problem solving (35).

There are further similarities to a quantitative study of Michaelis et al. (36) who described types of people with a mental illness and their preferences on shared decision-making. They differentiate between autonomous, collaborative, and passive types in terms of different predictive values. Participants with a high burden of depressive symptoms and low perceived self-efficacy withdrew into a passive treatment role (36). De Smet et al. (37) described this phenomenon as “being stuck between knowing vs. doing.”

Other studies only quantified the utilization of collaborative care and counted the care managers' patient contacts. For instance Grypma et al. (38) differentiate short-, intermediate-, and long-term patients, but they could not find a substantial difference in the development of the depressive symptoms. Nevertheless, the short-term patients reported fewer depressive episodes in their depression history. Here, they are comparable to the *active relief seekers* and the *active relaxation seekers* in GermanIMPACT. Even Petersen et al. (39) investigated symptom trajectories in the German PROMPT-Trial and identified two latent trajectories “fast improvers” and “slow improvers.” The main patient specific difference in this comparison was the severity in comorbidity, which was higher in the group of “slow improvers.” Nevertheless, even their developed typology of utilization could not identify any intervention-specific application, which is linked to an efficacious intervention.

### Types of Treatment Response

The qualitative study from Roberge et al. (40) supports the findings of our study. They asked physicians, nurses, social workers, and patients. Everyone identified comorbidities as a main obstacle in the therapy of depression (40). A systematic qualitative review on the implementation of collaborative care branded comorbidities as a main barrier as well (41). In an observational study with 792 patients, Rossom et al. (42) found that the subjective health status was the strongest hint for achieving remission. Not surprisingly, care managers and physicians in GermanIMPACT reported similarities in two further interview studies (43, 44).

Furthermore, our types of treatment response reflect the evidence of various studies. The severity of depression, the self-efficacy as well as the subjectively assessed coping behavior (45, 46), and less social support in general (47, 48) are strongly associated with the pathogenesis of depression and its success in different therapy settings.

### Patient-Specific Characteristics of the Types of Treatment Response Within in Types of Utilization

In the well-investigated field of factors that facilitate and hinder different therapy strategies against depression, our study adds the evidence in respect of the subjective perspective. The individual utilization of therapeutic elements, in relation to the development of depressive symptoms, largely depends on patient-specific characteristics. We could not identify similar studies that allowed such a concise evaluation of the effectiveness of a randomized controlled trial. However, as shown in the qualitative study from de Smet et al. (37), it is also true that positive changes such as dealing with insomnia and with other problems or an increased self-reflection could be stimulated in most cases. But the PHQ-9 measurement, which classifies our patients into *responders* and *non-responders*, is not sensitive to these effects.

To benefit in the outcome measurement of the current GermanIMPACT intervention, potential patients must have a recent history of depression with a low burden of comorbidities, and they should be able to implement pleasant activities.

### Recommendations for the IMPACT Intervention

To increase the effectiveness of the IMPACT intervention for a wider group of patients, upcoming interventions must be tailored to the patient-specific characteristics. *Non-responders* often suffered from so-called “early-onset depression,” a disease characterized by many negative patterns that solidified over time and a high burden of symptoms. Care managers should be enabled to deal with the specifics of chronic depression adequately. For example, elements of biographical work or techniques from the life review therapy (49) might be effective complementary methods.

The second step should be the consideration of common chronic mental and/or physical comorbidities, especially chronic pain, and anxiety. These comorbidities have been reported frequently by *non-responders*, passive problem-solving seekers, and passive relief seekers. On the one hand, disabling comorbidities were strong barriers to engaging in pleasant activities, while on the other hand, they increased the patients' suffering and contributed to their depressive symptoms. Many studies already demonstrated different approaches (50–53) and showed positive effects on depressive symptoms (54). For example, these approaches attempt to improve self-management skills in dealing with their chronic conditions, thereby reducing mental stress, increasing general activity, and finally improving the quality of life (55), and they should be used in the future.

Self-management skills are especially important. They are very closely related to self-efficacy and self-esteem in the responders' statements. Especially an increasing self-efficacy offers the chance to build up a sufficient self-esteem, which constitutes the basis for establishing and maintaining social contacts in our interviews.

Our non-responders with low self-esteem also complained about their social isolation, a small social network and loneliness. The key component of GermanIMPACT pleasant activations should be combined with intentional social engagement activities to reduce loneliness, as well as other symptoms of depression (19), to strengthen social support and to increase self-esteem (56–58).

### Strengths and Limitations

To the authors' knowledge, this is the first qualitative investigation of a collaborative care model for the treatment of depression, which investigated the quantifiable measured symptom history, combined with the subjective utilization of the intervention and the subjective perspective on the depressive disorder. Now further studies could provide statistical analyses of the relationship between types of utilization, types of treatment response, and patient characteristics.

The interviews were conducted after the intervention so that the survey and the therapy support could be perceived as independent. However, the retrospective consideration of the therapy support is critical because of subjective memory distortions (“recall bias”). That is why we choose the methodology of the problem-centered interview, which could produce more differentiated in-depth statements and valuable insights to adjust complex interventions (59).

Unfortunately, the intended sampling strategy could not be implemented regarding gender. No men were excluded, but we could ask only three eligible male participants. They were the only men who finished the GermanIMPACT intervention regularly. This illustrates the well-known fact that men could be hardly reached by health promotion programs (36), which might be due to social constructs of masculine values (60).

The results were generated in the context of the German health care system by using an intervention that has already tested itself internationally in different ways (18). Nevertheless, the German problems of time pressure in primary care, latency for specialized therapeutic care of depression, and self-stigmatization associated with special mental health care are international phenomena as well (61). Our typologies could support scientists, policy makers, and health care professionals to understand which types of patients will participate in such an intervention and in which manner they profit. Because of this, it may be possible to adapt the intervention according to the needs of patients and context. Even with the best adjustments to the GermanIMPACT intervention, it will be impossible to achieve remission for all patients (62). But older patients, who cannot benefit from a revised collaborative care of depressive disorders, could be sensitized for a more comprehensive therapeutic treatment. In this way, collaborative care could effectively support GPs as well as specialized care (40). Even in its current arrangement, it could promote the comprehensive integration of mental health into primary care demanded by the World Organization of National Colleges, Academies and Academic Associations of General Practitioners/Family Physicians, and the WHO, WONCA (63).

## Conclusion

Our two typologies emphasize that GermanIMPACT, in its current form as a low-threshold intervention, is suitable for moderately burdened patients with a depressive disorder. Combined, the two typologies show that an effective intervention requires a match between intervention components and patient needs. We saw no intersections between the types of utilization and treatment response. The typology of utilization did not suggest any specifications within the categories that contribute to an effective intervention. According to the types of treatment response, the patient-specific characteristics are crucial. An expansion of the intervention, especially for depression with a long history and comorbidities impairing mobility, could increase the effectiveness and improve the care situation of older people suffering from depression.

## Data Availability Statement

The datasets presented in this article are not readily available because the transcripts of the qualitative interviews cannot be made completely anonymous due to their level of detail. Requests to access the datasets should be directed to Thomas Kloppe, t.kloppe@uke.de.

## Ethics Statement

The studies involving human participants were reviewed and approved by Ethics Committee of the Medical Association of Hamburg. The patients/participants provided their written informed consent to participate in this study.

## Author Contributions

TK: conception and development of study design, coordinating contribution to data collection, contribution to data-analysis and interpretation, and main investigator responsible for data-analysis and interpretation. NP: contribution to data-analysis and interpretation and main reviewer of the manuscript. MS: contribution to conception and development of study design, contribution to data-analysis and interpretation, and reviewer of the manuscript. All authors contributed to the article and approved the submitted version. All authors contributed to the article and approved the submitted version.

## Conflict of Interest

The authors declare that the research was conducted in the absence of any commercial or financial relationships that could be construed as a potential conflict of interest.
